# Absent Arm Swing and Dual Tasking Decreases Trunk Postural Control and Dynamic Balance in People With Parkinson's Disease

**DOI:** 10.3389/fneur.2020.00213

**Published:** 2020-04-17

**Authors:** Tarique Siragy, Julie Nantel

**Affiliations:** School of Human Kinetics, University of Ottawa, Ottawa, ON, Canada

**Keywords:** postural control, trunk sway, dynamic balance, arm swing, Parkinson's disease, walking stability

## Abstract

**Introduction:** Falling during walking is a common occurrence in people with Parkinson's disease and is closely associated with severe social and medical consequences. Recent evidence demonstrates that arm swing affects dynamic balance in healthy young adults; however, it remains unexamined what its effect is in people with Parkinson's disease, particularly when combined with a secondary dual task.

**Methods:** Twenty people with Parkinson's disease (63.78 ± 8.97) walked with two arm swing conditions (absent and normal) with and without a secondary dual task. Data were collected on a split-belt treadmill CAREN Extended-System (Motek Medical, Amsterdam, NL). Average and standard deviations for trunk linear and angular velocity were calculated along with their instantaneous values (during foot strikes) in all three axes. Averages and coefficient of variations for step length, time, and width; margin of stability; and harmonic ratios were also calculated.

**Results:** Compared with normal arm swing, absent arm swing reduced the least affected leg's average step length and increased its step length coefficient of variation while increasing step time coefficient of variation in the most affected leg. Further, absent arm swing reduced trunk anteroposterior instantaneous angular velocity (least affected leg) and reduced anteroposterior instantaneous linear velocity (bilaterally). For the vertical axis, absent arm swing increased the trunk's average angular velocity but reduced its instantaneous linear velocity and angular velocity standard deviation (least affected leg). Additionally, the margin of stability increased when the arms were absent (least affected leg). Alternatively, dual tasking reduced average step time (most affected leg) and increased the step width coefficient of variation (bilaterally). Additionally, dual tasking increased the mediolateral average angular velocity, instantaneous linear velocity standard deviation (bilaterally), and instantaneous angular velocity standard deviation (least affected leg). For the vertical axis, dual tasking increased average linear and angular velocity standard deviation as well as instantaneous angular velocity standard deviation (bilaterally).

**Conclusion:** Findings suggest that participants attempted to control extraneous trunk movement (due to absent arm swing) through compensatory responses in both lower and upper extremities. However, participants appeared to predominately compensate on their least affected side. Contrastingly, modifying mediolateral foot placement appeared to be the main means of maintaining walking stability while dual tasking.

## Introduction

Parkinson's disease (PD) is the second most common neurodegenerative disease worldwide and is caused by progressive neurodegeneration within the basal ganglia of dopaminergic neurons (1, 2) In addition to causing PD's cardinal symptoms (bradykinesia, rigidity, and tremor), the basal ganglia's impaired function disrupts the gait pattern in people with PD (pwPD) (1). This is particularly concerning, as falling during walking is a debilitating threat that is closely associated with reduced autonomy and quality of life, hip fractures, and morbidity (3, 4).

Current evidence demonstrates that gait impairments in the lower extremity for pwPD include shorter stride length, increased stance time, increased spatiotemporal variability, and reduced interlimb coordination compared with those in healthy elderly adults (5–11). Although previous work has examined how PD affects postural control during static conditions and gait initiation, the quantification of postural control in pwPD during steady-state walking is not as prevalent (12–17). However, initial evidence demonstrates that pwPD walk with reduced peak trunk velocity in the frontal and sagittal planes in respect to age-matched adults (18). As the center of mass (COM) is located in the upper extremity and the lower extremity adapts foot placement to modify the base of support, each is an integral component for maintaining dynamic balance (defined as maintaining the COM within a moving base of support) (19, 20).

Traditionally, dynamic balance and rehabilitation research in pwPD are based on the inverted pendulum model, which proposes that arm swing passively arises from trunk motion, gravity, and inertia (21, 22). However, electromyography (EMG) and inverse dynamic evidence demonstrate that an active arm swing component assists in controlling trunk angular motion about the vertical axis (22). Although this improves gait's metabolic efficiency, research is conflicting whether arm swing affects dynamic balance and trunk postural control (22–24). Indeed, it is proposed that dynamic balance is enhanced either by arm swing facilitating a stable COM trajectory or by absent arm swing concentrating upper extremity mass, thereby increasing inertia (23, 24). Furthermore, it remains unexamined how arm swing affects trunk postural control and dynamic balance in pwPD. As reduced arm swing occurs in pwPD and intensifies as the disease progresses until completely absent, determining arm swings' effects holds direct implications for fall prevention in pwPD (25).

However, the comprehensive quantification of dynamic balance requires multiple metrics as each reflects a distinct aspect of gait's neuromuscular control (19). Indeed, neuromuscular control of gait requires input from both supraspinal and subcortical structures as well as the integration of feedback from a complex peripheral sensorimotor network (19, 26, 27). This multilayered neuromuscular control is necessary, as the upper and lower extremities have distinct movement patterns while walking (19, 28, 29). Bauby and Kuo were the first to demonstrate that gait parameters in the anteroposterior (AP) direction were controlled by “passive” automated neuromuscular control, whereas mediolateral (ML) parameters required “active” information processing (30). These mechanisms serve to not only control stability locally of an anatomical segment but also work in a coordinated manner to maintain global stability to avert a fall. As each dynamic balance metric is dependent on the trajectory and physical properties of the anatomical segment quantified, the direction of motion, time point in the gait cycle examined, and formulaic computation, no single metric is capable of quantifying dynamic balance in its entirety (19, 31). Thus, multiple metrics should be used simultaneously to quantify information that may be left unexamined by the use of any single metric.

Additionally, this multifaceted neuromuscular control provides a means for compensation should any aspect of the network become impaired (19, 26). For instance, in pwPD, dopamine loss impairs subcortical pathways responsible for gait automaticity and timing (8, 19, 32, 33). Thus, to compensate, pwPD recruit higher supraspinal structures to bypass the impaired subcortical pathways and direct additional attention for effective ambulation (5, 26). However, in ever-changing environments where multitasking is commonplace (terrain navigation, reading signs, talking, etc.), attention becomes divided between multiple concurrent tasks (34). When attention is divided between simultaneous tasks, the resources necessary to compensate for impaired gait automaticity become strained, thereby impairing effective locomotion (26). Although divided attention is demonstrated to impair lower extremity measures of dynamic balance in pwPD, its effect on additional dynamic balance metrics remains to be examined, particularly in the presence of varying arm swing conditions (26).

Therefore, our study's purpose was to examine normal and absent arm swing's effect on linear and angular trunk velocities as well as lower and upper extremity measures of dynamic balance with and without a dual task (DT) in pwPD. We hypothesize that absent arm swing and dual tasking will decrease instantaneous trunk linear and angular velocities as well as variability but increase these parameters' average values. Further, both arm swing and dual tasking will elicit unique responses from dynamic balance measures. Additionally, we predict that absent arm swing while dual tasking will be more destabilizing than all other conditions. Alternatively, walking with normal arm swing and dual tasking will only be more destabilizing compared with walking with normal arm swing without the DT.

## Methods

### Participants

Twenty pwPD (13 males and 7 females), aged 48–79 years (63.78 ± 8.97), were recruited from the Ottawa-Gatineau region. Participants were assessed with the original Unified Parkinson's Disease Rating Scale Motor examination (11 ± 6) and were between I and III on the Hoehn & Yahr scale. Average disease duration (8.0 ± 5.1) and age at onset (56.8 ± 9.60) data were collected. Further, seven participants reported freezing of gait based on the Freezing of Gait Questionnaire. However, because two participants presented severe dyskinesia and one participant having an incomplete session, only 17 participants were used in the analyses. As pwPD who develop dyskinesia are characterized by excessive movement dyscontrol, we excluded these participants from further analyses for our sample to be more representative of PD. Participants were tested on their optimally medicated state. An *a priori* power analysis revealed that 12 participants were adequate to achieve power at β = 0.8. Prior to data collection, volunteers were excluded if they reported any physical discomfort using a virtual reality system, reported any injuries and/or orthopedic surgeries that could interfere with gait, could walk only with the use of a walking aid, and had any additional illnesses other than PD. All participants provided written informed consent, and the study was approved by local ethics and scientific committees.

### Procedures

Participants walked with two arm swings (absent and normal) during single task (ST) and DT conditions for a total of four trials. ST trials lasted 3 min each, whereas DT trials were 2 min each. To assure safety and prevent falls, participants wore a safety harness attached to an overhead structure at all times. Arm conditions were randomized per block with all ST trials occurring before DT trials. The DT consisted of a word searching task with 12 familiar words randomly appearing in the participants' visual field. Words appeared one at a time on both left and right sides of a screen in front of participants and varied between 20 and 70°. Each word was shown for 3 s with a 2- to 4-s pause between subsequent words. Participants verbally called out each word as they appeared. During the absent arm swing trials, participants inserted their arms inside the safety harness, which effectively prevented arm motion. Participants were allowed to rest whenever necessary to minimize fatigue.

**Figure 1:**
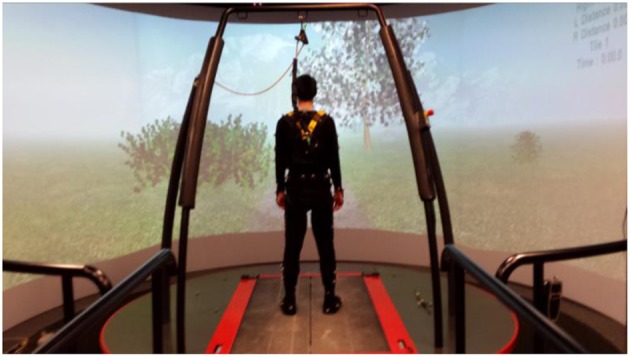
Experimental setup for the CAREN system virtual environment.

A 3D motion analysis was completed with the CAREN-Extended System (Motek Medical, Amsterdam NL) using a virtual park terrain. This system combines a 6°-of-freedom motion platform with embedded dual-belt treadmill, 12 Camera Vicon motion capture system, 180° projector screen, and a safety harness (Figure 1). The dual belt was synchronized so that both belts were symmetrically set at the participants' preferred walking speed. Three markers placed in the periphery of the treadmill were used to track platform motion, and a 57-marker set was used for tracking full body kinematics (31, 35). Kinematic data were collected at 100 Hz and ground reaction forces (GRFs) at 1,000 Hz.

### Kinematic and Kinetic Analyses

Markers and GRF data were processed in Vicon Nexus (Nexus 2.6, Oxford, UK), while 3D kinematics and kinetics were calculated in Visual 3D. A fourth-order, low-pass Butterworth filter with a 10-Hz cutoff frequency was used to filter marker data. To remove start-up effects, the first 25 s were removed before data analysis. Data were analyzed by custom Matlab scripts (MathWorks, Natick, MA) to calculate average trunk linear and angular velocities (average LV and AV, respectively) and variability (LV-SD and AV-SD, respectively) throughout the gait cycles as well as instantaneous velocities (instantaneous LV and AV, respectively) and velocity variability (instantaneous LV-SD and AV-SD, respectively) at heel strike. As the lower extremity is accepting the trunk's weight over a relatively small support at heel strike, quantifying instantaneous velocities provides potential subtleties overshadowed by average values (28). Additionally, average spatiotemporal parameters (step time, length, and width) and dynamic balance measures including the coefficient of variation (COV), margin of stability (MOS), and harmonic ratios (HRs) were quantified. The COV was calculated as follows:

$$\begin{array}{l} COV=\left(\frac{SD}{average}\right)x100 \end{array}$$

where SD is the standard deviation for the spatiotemporal parameter. The MOS was calculated bilaterally at both heel strikes and defined as the distance of the extrapolated COM (xCOM) to the right/left lateral heel marker.

$$\begin{array}{l} MOS=Lateral\;Heel\;Marker-xCOM \end{array}$$

The formula for xCOM was as follows:

$$\begin{array}{l} xCOM\;=COMp+\left(\frac{COMv}{\omega_{\Theta }}\right) \end{array}$$

where COM_p_ = COM's position and COM_v_ = COM's velocity. ϖ_Θ_ was calculated as follows:

$$\begin{array}{l} \omega_{\Theta }=\surd g/l \end{array}$$

In this term, *g* = 9.81 m/s^2^ and *l* is the length of the inverted pendulum determined as the average distance of the right/left lateral heel marker to the COM at heel strikes. Visual 3D was used to calculate the COM's position and velocity. The MOS was only calculated in the ML direction, as this metric is only valid in this direction during steady-state walking (31, 36).

HRs were calculated on the center of gravity's (COG's) acceleration defined as the first central difference of the COG's velocity. The HR examines a signal's periodicity by calculating a ratio of the amplitudes of the even and odd harmonics obtained through a fast Fourier analysis. The HR for the AP (HR-AP) and VT (HR-VT) directions were calculated as the first 10 even harmonics divided by the first 10 odd harmonics, whereas the HR-ML calculation was the inverse, and higher values in all cases indicated greater dynamic balance (19, 31, 37–39). All discrete metrics were quantified at heel strikes for the least and most affected legs.

### Statistical Analyses

Data were analyzed using SPSS 23.0, and *p* < 0.05 was considered statistically significant. The normality of variables was verified using Shapiro–Wilk's test, and a two-way repeated measure ANOVA was performed to find the effect of arm swing, dual tasking, and potential interactions. If statistical significance was achieved with the ANOVA (*p* < 0.05), then pairwise comparisons with a Sidak–Bonferroni adjustment for multiple comparisons were used for *post-hoc* analyses. After the correction, findings were only deemed statistically significant when *p* < 0.026.

## Results

Dynamic balance (HRs, MOS, and COV) measures are reported in Table 1A, whereas spatiotemporal averages are reported in Table 1B. Average trunk linear and angular velocities as well as variabilities are reported in Table 2A, whereas their instantaneous values at heel strike are in Table 2B.

**Table 1A:** Dynamic balance measures for arm (absent and normal) and task (single and dual) conditions: coefficient of variation for step length, width, and time; harmonic ratios in all three axes; and mediolateral margin of stability.

		**Single task**		**Dual task**		***P*-value**
		**Normal**	**Absent**	**Normal**	**Absent**	
Harmonic ratios	AP	3.07 ± 1.03	2.93 ± 0.88	3.03 ± 0.87	3.16 ± 0.84	0.956
	ML	5.16 ± 0.94	5.30 ± 1.25	5.07 ± 1.28	5.13 ± 1.31	0.640
	Vert	3.97 ± 1.87	3.53 ± 1.71	3.54 ± 1.23	3.63 ± 1.29	0.135
Margin of stability (cm)		11.40 ± 1.2	11.79 ± 1.4	11.39 ± 1.49	11.73 ± 1.69	0.018^†^
		11.09 ± 1.87	11.35 ± 1.88	11.04 ± 1.97	11.24 ± 2.12	0.117
COV step length		3.69 ± 1.62	4.73 ± 2.36	4.19 ± 1.95	4.15 ± 1.64	0.015^†^
		3.96 ± 1.19	4.54 ± 2.29	4.40 ± 1.89	4.43 ± 1.39	0.269
COV step time		2.47 ± 0.54	3.09 ± 1.04	3.06 ± 1.03	3.00 ± 0.68	0.152
		2.61 ± 0.58	3.33 ± 1.37	2.89 ± 0.89	2.94 ± 0.73	0.027^†^
COV step width		8.45 ± 3.16	8.34 ± 3.91	10.19 ± 3.92	10.00 ± 4.39	0.690^*^
		8.04 ± 2.00	8.47 ± 3.14	10.13 ± 3.85	9.70 ± 3.89	0.985^*^

*Coefficient of variation and margin of stability were quantified at foot strike of the least and most affected legs. p-values from the two-way repeated measures ANOVA are reported for arm swing main effects*.

† *Arm swing main effects at p < 0.05*.

* *Dual-task main effects at p < 0.05*.

**Table 1B:** Averages for step length, time, and width for arm (absent and normal) and task (single and dual) conditions at foot contact for least (white) and most (gray) affected legs.

	**Single task**		**Dual task**		***p*-value**
	**Normal**	**Absent**	**Normal**	**Absent**	
Average length (cm)	50.30 ± 6.78	48.11 ± 6.89	49.73 ± 6.82	49.25 ± 6.14	0.017^†^
	48.96 ± 6.76	46.93 ± 6.85	47.82 ± 6.31	47.86 ± 6.11	0.057
Average time (ms)	554 ± 54	554 ± 61	542 ± 56	541 ± 52	0.861^*^
	538 ± 39	536 ± 45	536 ± 41	530 ± 44	0.155
Average width (cm)	18.99 ± 3.96	19.56 ± 4.36	18.91 ± 4.52	19.46 ± 4.73	0.071
	18.94 ± 3.98	19.31 ± 4.23	18.99 ± 4.47	19.36 ± 4.71	0.206

*p-values from the two-way repeated measures ANOVA are reported for arm swing main effects*.

† *Arm swing main effects at p < 0.05*.

* *Dual-task main effects at p < 0.05*.

**Table 2A:** Trunk average linear and angular velocities and variabilities in all three axes for arm (absent and normal) and task (single and dual) conditions.

		**Single task**		**Dual task**		***p*-value**
		**Normal**	**Absent**	**Normal**	**Absent**	
Trunk linear velocity (cm/s) (×10^−2^)	AP	22 ± 3	13 ± 20	14 ± 26	18 ± 24	0.407
	ML	15 ± 3	14 ± 8	13 ± 10	17 ± 11	0.420
	Vert	−15 ± 4	−14 ± 5	−15 ± 4	−15 ± 5	0.178
Trunk linear velocity variability (cm/s) (×10^−2^)	AP	312 ± 90	327 ± 127	377 ± 181	333 ± 106	0.302
	ML	201 ± 53	199 ± 66	251 ± 64	254 ± 69	0.923^*^
	Vert	31 ± 10	32 ± 12	38 ± 18	35 ± 12	0.482^*^
Trunk angular velocity (°/s) (×10^−2^)	AP	12 ± 10	11 ± 11	13 ± 9	13 ± 11	0.797
	ML	4 ± 14	5 ± 12	6 ± 13	7 ± 15	0.271^*^
	Vert	6 ± 8	12 ± 8	7 ± 8	10 ± 11	0.011^†^
	AP	92 ± 24	95 ± 25	105 ± 40	99 ± 35	0.614
Trunk angular velocity variability (°/s) (×10^−2^)	ML	80 ± 22	78 ± 18	94 ± 30	91 ± 22	0.421^*^
	Vert	158 ± 51	153 ± 50	226 ± 67	219 ± 66	0.321^*^

*Values were rounded to the nearest whole number. p-values from the two-way repeated measures ANOVA are reported for arm swing main effects*.

† *Arm swing main effects at p < 0.05*.

* *Dual-task main effects at p < 0.05*.

**Table 2B:** Instantaneous trunk linear and angular velocities and variabilities in all three axes for arm (absent and normal) and task (single and dual) conditions at heel strike of least (white area) and most affected (gray area) legs.

		**Single task**		**Dual task**		***p*-value**
		**Normal**	**Absent**	**Normal**	**Absent**	
Inst. trunk linear velocity (cm/s)	AP	4.7 ± 1.8	3.2 ± 1.6	4.5 ± 2.6	3.7 ± 1.9	0.004^†^
		4.0 ± 2.9	3.0 ± 2.1	3.8 ± 2.9	3.1 ± 2.4	0.016^†^
	ML	−1.7 ± 15.4	−1.3 ± 15.3	−1.3 ± 15.3	−0.8 ± 15.3	0.062
		1.3 ± 15.8	1.2 ± 15.5	1.0 ± 16.0	1.0 ± 15.8	0.852
	Vert	−17.0 ± 4.0	−15.4 ± 5.0	−16.7 ± 10.0	−15.7 ± 5.0	0.002^†^
		−15.3 ± 3.9	−13.7 ± 4.2	−14.8 ± 4.0	−15.2 ± 4.7	0.069
Inst. trunk linear velocity variability (cm/s)	AP	3.5 ± 0.9	3.6 ± 1.1	3.8 ± 1.4	3.5 ± 1.0	0.568
		3.4 ± 1.0	3.5 ± 1.1	3.8 ± 1.4	3.6 ± 1.2	0.829
	ML	2.7 ± 0.5	2.5 ± 0.7	3.1 ± 0.8	3.1 ± 0.7	0.491^*^
		2.4 ± 0.5	2.5 ± 0.6	2.9 ± 0.7	2.9 ± 0.6	0.228^*^
	Vert	16.8 ± 0.6	18.2 ± 0.7	18.6 ± 0.8	19.5 ± 0.6	0.191
		18.4 ± 0.6	18.4 ± 0.6	18.4 ± 0.6	19.3 ± 0.6	0.544
Inst. trunk angular velocity (°/s)	AP	6.1 ± 5.2	3.9 ± 6.5	6.0 ± 6.2	4.5 ± 7.4	0.008^†^
		6.6 ± 4.5	4.3 ± 4.4	7.1 ± 4.8	4.6 ± 5.6	0.005^†^
	ML	−0.2 ± 6.0	−0.7 ± 7.7	−0.9 ± 7.8	−0.9 ± 7.9	0.844
		0.5 ± 4.7	0.8 ± 5.7	2.1 ± 5.4	1.4 ± 6.9	0.782
	Vert	−2.9 ± 10.5	−3.1 ± 17.6	−3.1 ± 11.3	−3.9 ± 18.2	0.828
		1.4 ± 11.4	4.2 ± 14.6	3.1 ± 14.0	3.5 ± 17.0	0.336
Inst. trunk angular velocity variability (°/s)	AP	3.8 ± 1.2	3.8 ± 1.0	4.1 ± 1.0	4.0 ± 1.3	0.506
		3.5 ± 1.1	3.7 ± 1.0	3.7 ± 1.0	4.0 ± 1.1	0.131
	ML	2.7 ± 1.0	2.5 ± 0.5	2.8 ± 1.0	2.9 ± 0.6	0.620^*^
		2.7 ± 1.0	2.6 ± 0.6	2.7 ± 0.8	2.9 ± 1.1	0.523
	Vert	5.8 ± 2.0	5.1 ± 2.2	6.5 ± 2.3	5.9 ± 1.9	0.051^*^
		5.7 ± 1.6	4.7 ± 1.2	6.1 ± 2.1	5.7 ± 1.6	0.025^†^

*p-values from the two-way repeated measures ANOVA are reported for arm swing main effects*.

† *Arm swing main effects at p < 0.05*.

* *Dual-task main effects at p < 0.05*.

### Arm Swing

ANOVA results revealed that absent arm swing elicited the following responses in the least affected leg: a reduced average step length [*F*_(1, 16)_ = 7.06, *p* = 0.017, $\eta_{\text{p}}^{2}$ = 0.306] and increased COV step length [*F*_(1, 16)_ = 7.48, *p* = 0.015, $\eta_{\text{p}}^{2}$ = 0.319]. Further, absent arm swing increased the most affected leg's step time COV [*F*_(1, 16)_ = 5.90, *p* = 0.027, $\eta_{\text{p}}^{2}$ = 0.269]. Additionally, an arm-task interaction occurred for COV step length in the least affected leg where the ST normal arm swing had less variability than the ST absent arm swing [*F*_(1, 16)_ = 6.08, *p* = 0.025, $\eta_{\text{p}}^{2}$ = 0.275]. A further interaction existed for the least affected leg's COV step time where ST normal arm swing had less variability than dual-task absent arm swing [*F*_(1, 16)_ = 5.21, *p* = 0.037, $\eta_{\text{p}}^{2}$ = 0.245].

In the AP direction, absent arm swing reduced the trunk's instantaneous LV at heel strike of the least [*F*_(1, 16)_ = 11.314, *p* = 0.004, $\eta_{\text{p}}^{2}$ = 0.414] and most [*F*_(1, 16)_ = 7.217, *p* = 0.016, $\eta_{\text{p}}^{2}$ = 0.311] affected legs. Additionally, the trunk's instantaneous AV decreased without arm swing at heel strike of the least affected leg [*F*_(1, 16)_ = 9.161, *p* = 0.008, $\eta_{\text{p}}^{2}$ = 0.364]. Alternatively, in the ML direction, absent arm swing increased the MOS [*F*_(1, 16)_ = 7.00, *p* = 0.018, $\eta_{\text{p}}^{2}$ = 0.304] at heel strike for the least affected leg. Along the vertical axis, absent arm swing reduced the trunk's instantaneous LV at heel strike of the least affected leg [*F*_(1, 16)_ = 13.831, *p* = 0.002, $\eta_{\text{p}}^{2}$ = 0.464]. Further, absent arm swing increased average trunk AV [*F*_(1, 16)_ = 8.37, *p* = 0.011, $\eta_{\text{p}}^{2}$ = 0.343] and reduced the instantaneous trunk AV-SD at heel strikes of the least [*F*_(1, 16)_ = 4.45, *p* = 0.051, $\eta_{\text{p}}^{2}$ = 0.218] and most [*F*_(1, 16)_ = 8.740, *p* = 0.025, $\eta_{\text{p}}^{2}$ = 0.276] affected legs. An arm-task interaction also occurred for instantaneous trunk LV at heel strike of the most affected leg (*p* = 0.031), but *post-hoc* tests were non-significant.

### Dual Task

For dual-tasking main effects, walking with the secondary task reduced the least affected leg's average step time [*F*_(1, 16)_ = 6.117, *p* = 0.025, $\eta_{\text{p}}^{2}$ = 0.277]. Dual-task walking also increased the step width COV for the least [*F*_(1, 16)_ = 9.252, *p* = 0.008, $\eta_{\text{p}}^{2}$ = 0.366] and most [*F*_(1, 16)_ = 10.321, *p* = 0.005, $\eta_{\text{p}}^{2}$ = 0.392] affected legs. In the ML direction, dual tasking increased the trunk's average AV [*F*_(1, 16)_ = 4.793, *p* = 0.044, $\eta_{\text{p}}^{2}$ = 0.230], AV-SD [*F*_(1, 16)_ = 6.748, *p* = 0.016, $\eta_{\text{p}}^{2}$ = 0.297], and LV-SD [*F*_(1, 16)_ = 15.947, *p* = 0.001, $\eta_{\text{p}}^{2}$ = 0.499]. Further, DT increased the trunk's instantaneous LV-SD at the heel strikes for the least [*F*_(1, 16)_ = 9.335, *p* = 0.008, $\eta_{\text{p}}^{2}$ = 0.368] and most [*F*_(1, 16)_ = 12.550, *p* = 0.003, $\eta_{\text{p}}^{2}$ = 0.440] affected legs as well as the trunk's instantaneous AV-SD for the least affected leg [*F*_(1, 16)_ = 5.90, *p* = 0.027, $\eta_{\text{p}}^{2}$ = 0.269]. Furthermore, the trunk's LV-SD [*F*_(1, 16)_ = 6.555, *p* = 0.021, $\eta_{\text{p}}^{2}$ = 0.291] and AV-SD [*F*_(1, 16)_ = 12.746, *p* = 0.003, $\eta_{\text{p}}^{2}$ = 0.443] increased throughout the entire gait cycle while dual tasking. Additionally, dual tasking increased the trunk's instantaneous AV-SD at heel strike of the least [*F*_(1, 16)_ = 7.28, *p* = 0.016, $\eta_{\text{p}}^{2}$ = 0.313] and most [*F*_(1, 16)_ = 9.078, *p* = 0.008, $\eta_{\text{p}}^{2}$ = 0.362] affected leg.

## Discussion

### Main Findings

This study examined how absent and normal arm swing affects dynamic balance and postural control while walking with and without a DT in pwPD. Our results supported our hypothesis that each dynamic balance measure would respond uniquely to absent arm swing and DT. Indeed, absent arm swing increased the MOS and step length COV for the least affected leg as well as increased step time COV in the most affected leg. However, absent arm swing did not affect the HR. Alternatively, DT increased step width COV for both legs but did not affect the MOS or HR values. Further, our hypothesis on trunk postural control was only partially supported. Indeed, absent arm swing only increased average AV about the vertical axis. However, as predicted, absent arm swing decreased instantaneous LV and AV in the AP direction as well as decreased instantaneous AV-SD about the vertical axis. Alternatively, DT increased average AV only in the ML direction as well as increased average and instantaneous variabilities only in the ML direction and vertical axis.

### Arm Swing

Our hypothesis that absent arm swing would increase trunk velocity was only partially supported. Indeed, only the average trunk AV about the vertical axis increased when arm swing was removed. This was expected as the 1:1 arm–leg swing ratio controls the trunk's angular motion about the vertical axis by equalizing the torques acting on the COM (22). However, as hypothesized, instantaneous AP-LV and VT-LV and AP-AV decreased during absent arm swing, suggesting that our participants adopted a compensatory trunk stiffening strategy. Previous research demonstrates that pwPD adopt this strategy to control excessive trunk movement during dynamic movements such as walking (18). These changes in instantaneous velocities at heel strike, particularly in the AP direction, may be due to the importance that this time point has in the gait cycle as it marks the beginning of interlimb trunk transfer (28, 29). Our participants may have reduced the instantaneous AP-LV to reduce the likelihood of a forward balance loss or the need for a reactive recovery step while the trunk is transferred between limbs. Additionally, at heel strike, the trunk flexes forward under the force of gravity but is attenuated by an opposing force from lower back and hip musculature (28, 29). Therefore, the reduced instantaneous AP-AV suggests that our participants adopted a stiffening strategy to maintain a more erect posture to attenuate gravity's perturbing effect.

Although a trunk stiffening strategy accounts for our AP and VT findings, it does not account for the lack of changes in the ML direction. This disparity likely arose due to the different postural strategies observed for trunk AP and ML movement in pwPD (40). Additionally, Jehu and Nantel discussed that although a stiffening strategy enhances postural control in the short term, it could also lead to an inability to efficiently implement adaptive responses to internal and external perturbations (18). Thus, our participants may not have restricted ML mobility to appropriately adapt to the increased trunk rotation during absent arm swing. Preserving ML mobility would also account for the increased MOS when the arms were absent. By increasing the xCOM distance to the base of support's edge, our participants enhanced their ability to mitigate global balance loss in the ML direction despite the faster rotating trunk (41, 42).

Interestingly, increases in our participants' MOS only occurred at heel strike of the least affected side. In PD, asymmetric neurodegeneration compromises mobility in one leg to a greater degree than the other, causing an asymmetric gait (43–47). In an examination of asymmetric walking, Buurke et al. demonstrated that the MOS is larger in the faster leg in healthy adults (48). The authors discussed that this disparity arose either passively from the faster leg's reduced stance time (which increases the MOS) or actively as a compensatory response (48). In our study, no change in step time occurred during the absent arm condition, suggesting active adaptation. Therefore, our MOS findings likely resulted from the asymmetric neurodegeneration impairing the most affected leg's adaptation to absent arm swing. Although this strategy is intended to enhance global balance, it potentially could be maladaptive because the xCOM is now shifted closer toward the most affected leg. Should an external perturbation occur on the most affected leg, the xCOM would now have to overcome a greater distance to be in a position where the contralateral leg could act as a base of support. Similarly, asymmetric neurodegeneration would account for the reduction in only the least affected leg's step length. Huang et al. demonstrated that spinal rotational amplitudes decrease when healthy adults reduce their step length (49). Thus, our participants plausibly adapted their step length to attenuate the increased trunk rotation during absent arm swing. This may be a mechanism to decrease and partially counteract the torques acting on the COM's vertical angular motion, a task normally accomplished through contralateral arm–leg swing (22). However, lack of findings in the most affected leg suggests that this side was impaired in its ability to execute a similar response.

Additionally, adaptation differences between legs to the absent arm swing may account for our COV findings. Although large spatiotemporal variability is a strong predictor of falls in pwPD, previous research suggests that a certain amount of variability is necessary for lower extremity adaptation (19, 50). However, the COV only quantifies variability's magnitude and is therefore incapable of parsing adaptive responses from neuromuscular impairments (19, 33). The increases in step length COV for the least but not the most affected leg may partially be due to the aforementioned step length adaptation. Alternatively, the most affected leg's increased step time COV may indicate motor impairment in that leg's rhythmic temporal sequence. However, additional research is necessary to elucidate variability differences in lower limb motor adaptability and instability in pwPD. To this end, our interaction (least affected leg's: COV step length being lower during ST normal arm swing than ST absent arm swing and its COV step time being lower during ST normal arm swing than DT absent arm swing) results plausibly reflect the differences in variability that could arise from adaptation or from neuromuscular impairment. For instance, the interaction that occurred for the least affected leg's COV step length between ST conditions supports the notion that the changes in this parameter were an adaptive mechanism to partially counteract the increased trunk rotation. However, the COV step time interaction in the least affected leg is plausibly more indicative of neuromuscular impairment as the DT absent arm swing is arguably the most destabilizing condition. Further, as our participants were in the moderate stage of disease progression, more variable step timing may only have been elicited when the two destabilizing conditions (DT and absent arm swing) were combined. Differences in step time COV as a reflection of neuromuscular impairment holds with evidence demonstrating impairment to the internal timing of movement in pwPD (5, 26, 51). Unexpectedly, no HR differences were observed. HRs quantify dynamic balance by examining the COM's periodicity and reflect (a)symmetric leg motion (37, 38). As pwPD walk with increased spatiotemporal asymmetry, our findings suggest that absent arm swing did not affect our participants' gait asymmetry level (43).

Although our arm swing results demonstrate the contribution of nominal arm swing to dynamic balance and postural control in pwPD, which becomes asymmetric and absent with disease progression, consciously increasing arm swing may also elicit changes in gait parameters (25, 52). Indeed, empirical evidence demonstrated that increasing arm swing beyond nominal levels deteriorates dynamic balance and postural control in healthy young adults (31). This may be due to an internal focus of attention elicited when individuals are made to consciously modify their own mechanics and, similarly, a dual-tasking effect as individuals would direct their attention to the secondary task of moving their arms. As pwPD have a reduced DT ability due the disease's neurodegenerative nature, this demographic may have additional difficulty in consciously modifying and maintaining arm motion (5, 26). Thus, future work and clinicians should consider examining interventions that are more implicit and do not tax attentional resources to restore the contralateral arm–leg swing pattern.

### Dual Task

Our hypothesis that DT would increase trunk velocity and variability was supported as DT increased variability in the mediolateral and vertical axes. However, the additional increase in the ML average AV suggests that DT was more threatening to our participants' postural control in this direction. Mackinnon and Winter discussed that during walking, the neuromuscular system mitigates gravity's destabilizing torque in this direction through a counterbalancing torque generated by hip and trunk musculature (53). This careful balancing of torques maintains an individual's erect posture and leveled visual field (53). In healthy adults, precisely counterbalancing gravity's perturbing effects on the trunk is partially accomplished through proprioception (27). However, previous research indicates that this system is impaired relatively early in pwPD, which reduces their kinesthetic sense (27, 54, 55). Therefore, pwPD compensate by relying on visuomotor control, which remains largely spared from the disease's neurodegeneration as it is conveyed primarily through neuronal afferents to the cerebellum (27, 56). However, visuomotor feedback requires attentional resources from higher level cortical structures for active information processing (30). As such, our participants' increased frontal plane AV as well as AV-SD and LV-SD during DT plausibly arose as attention was divided between postural control and the visual word searching task. Interestingly, participants' instantaneous AV-SD in the ML direction only increased for the least affected leg. This may be due to the asymmetric neurodegeneration, limiting mobility and potential adaptive responses that could occur at heel strike on the most affected side.

Additionally, as trunk movement determines lower extremity foot placement, the increases in our ML trunk variability during DT accounts for the simultaneous increase in step width COV for both legs (28, 29, 53). Although large changes in step width COV indicate reduced dynamic balance, a certain amount of variability is necessary for foot placement adaptation to maintain ML balance (19, 50, 57). Therefore, when our step width COV results are examined alongside the MOS findings, our results suggest a more adaptive response whereby our participants correctly predicted the trunk's irregular ML trajectory and appropriately adapted foot placement to preserve their existing global dynamic balance (31). However, our findings conflict with previous reports, demonstrating no changes in step width COV in pwPD while dual tasking (50). This may be due to conflicting evidence demonstrating both increases and decreases in pwPD's postural sway compared with that of age-matched adults (58, 59). During walking, the magnitude of trunk sway directly influences ML foot placement; therefore, multiple responses to DT would elicit various responses in step width COV.

Various DT responses further account for our lack of findings in step time and length COV as well as HRs. Indeed, Yogev-Seligmann et al., discussed that DT responses in pwPD are dependent on task complexity and the stage of disease progression (26). In our study, the simple word searching task may not have challenged our participants' attentional resources to a degree that caused a breakdown in their rhythmic spatiotemporal control nor their level of gait asymmetry. Interestingly, our participants' lower extremity response to DT was somewhat unexpected, as step time was reduced in the least affected leg. This conflicts with evidence demonstrating that pwPD display a more “cautious gait” strategy when dual tasking, which includes an increase in step timing (5, 6, 26). However, previous research demonstrates that spatiotemporal asymmetry increases in pwPD during DT (9, 26). In our study, therefore, the least affected leg's reduced step time plausibly arose due to the DT progressively increasing our participants' gait asymmetry. In summary, our DT results indicate that clinicians should consider therapeutic programs that facilitate appropriate foot placement to adapt the base of support in pwPD to maintain global dynamic balance. Further, clinicians should carefully consider the complexity of the DT used in the therapy to be appropriate to the stage of PD progression and the phenotype of PD presented. This is particularly crucial as the amount of attentional resources available to control posture will vary, which impacts pwPD ability to maintain postural control in the ML direction during multitasking.

### Limitations

When considering our findings, it is important to consider that our participants were tested in the optimally medicated state. Empirical evidence on l-Dopa demonstrates that ML postural sway (displacement and velocity) deteriorates when pwPD are in their “on” medicated state compared with “off” (40). Further, our COV parameters would be impacted by the medication state, as medication reduces spatiotemporal variability in pwPD (11, 60). Additionally, differences between freezers and non-freezers were not examined, which may yield insightful nuances into the responses elicited in each group during absent arm swing (26, 47, 61). As neurodegeneration is suggested to be more widespread in freezers than non-freezers, the ability to execute adaptive responses to the DT and absent arm swing may be unique to each group (26). Finally, our results are limited in that no aged-matched control was used to compare the effects of arm swing and dual tasking. Future research should consider comparing pwPD with an aged-matched control group, as both groups may respond and compensate differently to absent arm swing and dual-tasking conditions.

## Conclusion

To conclude, removing arm swing in pwPD increased average trunk AV about the vertical axis and elicited compensatory responses in both the upper and lower extremities. These responses arose to compensate for the absent 1:1 arm–leg swing ratio that controls trunk angular motion about the vertical axis. Indeed, the trunk stiffening strategy in our participants was an upper extremity response to plausibly avert forward balance loss and maintain a more erect posture at heel strike when the arms were removed. Additionally, the alterations in the least affected leg (increased MOS, increased step length variability, and decreased average step length) suggest that pwPD attempt to enhance global dynamic balance and attenuate the increased trunk rotation as compensation for absent arm swing. However, PD's asymmetric neurodegeneration appears to impede the ability to bilaterally execute these strategies in response to internal perturbations such as the increased trunk rotation. Alternatively, the absent arm swing condition plausibly disrupted the most affected leg's temporal sequence as indicated by the increased step time variability. Our findings, therefore, point to the effective role that arm swing plays in maintaining dynamic balance in pwPD. As arm swing amplitude is reduced and asymmetric in pwPD, clinicians should consider programs that aim to restore gait's contralateral arm–leg swing ratio to enhance dynamic balance and postural control. However, caution must be executed in the therapeutic method used to restore nominal arm–leg swing motion, as consciously increasing arm swing while walking is demonstrated to negatively impact gait mechanics. Finally, therapeutic programs should incorporate strategies that maintain trunk postural control, particularly in the mediolateral direction, and facilitate lower extremity adaptation during DT. As multitasking is commonplace in everyday situations, strategies aimed at training postural control during DT would be beneficial in fall prevention for pwPD. Indeed, our results demonstrate that DT reduces pwPD ability to maintain postural control in the ML direction as indicated by the increase in our participants' average AV as well as AV-SD and LV-SD. Although postural control is an integral component that contributes to dynamic balance, our participants were able to avert a balance loss by adapting their ML foot placement to keep their xCOM within their base of support, thereby demonstrating that adaptation is partially preserved in pwPD.

## Data Availability Statement

The datasets generated for this study are available on request to the corresponding author.

## Ethics Statement

The studies involving human participants were reviewed and approved by University of Ottawa, Office of Research Ethics and Integrity. The patients/participants provided their written informed consent to participate in this study.

## Author Contributions

JN conceptualized and organized the research project. Data analysis was performed by TS (first analysis) and JN (review and critique). Statistical analysis was performed by TS (design and execution) and JN (design, review, and critique). TS wrote the first draft of the manuscript. JN reviewed and provided critical revision.

## Conflict of Interest

The authors declare that the research was conducted in the absence of any commercial or financial relationships that could be construed as a potential conflict of interest.
